# Editorial: Planctomycetes-Verrucomicrobia-Chlamydiae Bacterial Superphylum: New Model Organisms for Evolutionary Cell Biology

**DOI:** 10.3389/fmicb.2017.01458

**Published:** 2017-08-02

**Authors:** Laura van Niftrik, Damien P. Devos

**Affiliations:** ^1^Microbiology, Faculty of Science, Institute for Water and Wetland Research, Radboud University Nijmegen, Netherlands; ^2^Centro Andaluz de Biología del Desarrollo-CSIC, Universidad Pablo de Olavide Seville, Spain

**Keywords:** PVC bacteria, bioactive compounds, peptidoglycan, cell surface, genetic tools, cell biology and cell division

## Historical controversies in planctomycetes-verrucomicrobia-chlamydiae (PVC) research

The PVC superphylum bacteria have managed to intrigue and inspire researchers from the very start. Especially morphological and cell biological features of many PVC members puzzled, and in some cases, even misguided us researchers. This is illustrated by the initial misidentification of *Chlamydia trachomatis* as a virus (von Prowazek and Halberstadter, 1907), and the confusing etymology of Planctomycetes, meaning “floating fungus” (Gimesi, 1924). Toward the end of the last century, the controversy became even greater. The status of superphylum, the common ancestry of these bacteria with diverse genotypes, phenotypes, and life styles, was not yet recognized. In addition, the cell wall structure and apparent intracellular compartmentalization ran contrary to the classical bacterial dogma. Planctomycetes and Chlamydia were proposed to be devoid of peptidoglycan, an otherwise ubiquitous bacterial cell wall polymer (König et al., 1984; Liesack et al., 1986; McCoy and Maurelli, 2006; Cayrou et al., 2010). Planctomycetes were hypothesized to have a “third cell plan,” neither Gram-negative nor Gram-positive, and this was exemplified by *Gemmata obscuriglobus*, which was considered “the nucleated bacterium” (Fuerst, 2005). Further adding to the confusion was the observation that the Planctomycete undertaking anaerobic ammonium oxidation (anammox) did so by employing a specific intracellular anammoxosome compartment to support this process (Strous et al., 1999). The report of endocytosis-like protein uptake (previously only observed in eukaryotes) in *G. obscuriglobus* (Lonhienne et al., 2010) added more controversy and eventually it obtained the status of the “Platypus of microbiology” (Devos, 2013).

Only at the start of the present century, light began to dawn on this conundrum. First, commonalities of characters and phylogenies converged to the recognition of the PVC superphylum (Wagner and Horn, 2006). Then, the hypothesis that they represent variations of, but no exception to, the Gram-negative cell plan was percolating most research and discussions (Devos, 2014). This eventually culminated with the discovery of peptidoglycan in almost all PVC members investigated (Pilhofer et al., 2013; Liechti et al., 2014; Jeske et al., 2015; van Teeseling et al., 2015; and this Research topic).

Since then, the PVC research community began to catch momentum and genome data and publication rate have increased exponentially. With the superphylum status now amply accepted, it is clear that PVC bacteria are fascinating new model organisms for bacterial and evolutionary cell biology. PVC bacteria are relevant to the environment (they are found in most sampled environments and are important contributors to major biogeochemical cycles), biotechnology (they are potential producers of bioactive compounds and used in bioremediation as well as responsible for the anammox process which is applied in wastewater treatment), evolutionary cell biology (they have features that separate them from other bacteria, such as extensive bacterial endomembrane systems and atypical modes of cell division) and human health (their presence has been linked to various conditions, from obesity to developmental disorders; Devos and Ward, 2014). The articles presented in this Research topic are a reflection of this diversity of research on PVC bacteria.

## Bioactive compounds

Marine environments are a source of bioactive compounds. Many of these bioactive compounds are derived from sponges and macroalgae and their associated microbiome. Interestingly, some of the microbiomes have been reported to contain a high number of the slow-growing Planctomycetes. Therefore, there is currently a high interest in the potential of Planctomycetes as sources of bioactive compounds and antibiotics. Vollmers et al. investigated a brown macro-algae biofilm (*Macrocystis pyrifera*) from Monterey Bay for Planctomycetes and Verrucomicrobia using metagenomic shotgun and amplicon sequencing. Novel species were found and all contained secondary metabolite-related gene clusters. Jeske et al. developed a pipeline to cultivate and screen Planctomycetes for the production of antimicrobial compounds and showed antimicrobial activity of extracts from three Planctomycete species. Graça et al. isolated 40 Planctomycetes from macroalgae from the Portuguese coast and screened them for the production of antimicrobial compounds using molecular analysis (non-ribosomal peptide synthase and polyketide synthase genes) and bioactivity assays. The majority of the screened Planctomycetes (95%) contained one or both classes of secondary metabolite genes. In addition, approximately half of the Planctomycete extracts had antifungal (against *Candida albicans*, 43%) and antibacterial (against *Bacillus subtilis*, 54%) activity.

## New PVC genera and species needed

Despite their relevance in global nutrient cycles, industrial applications, human health and evolution, the Planctomycetes, and Verrucomicrobia phyla are still largely undersampled. Kohn et al. isolated a novel Planctomycete strain from the Wadden Sea (Germany) that is phylogenetically distant from other Planctomycetes and represents a novel genus. The isolate has an exceptionally large genome (9 Mb) including 45 “giant-genes.” They named the new Planctomycete after one of the Planctomycete research pioneers: Prof. John Fuerst (*Fuerstia marisgermanicae*).

## Cell biology

As described earlier, the PVC cell wall was a controversial topic for quite some years. Recent work has refuted the absence of peptidoglycan for both Chlamydia and Planctomycetes and in this Research topic, the lack of peptidoglycan is also challenged for Verrucomicrobia. Naqvi et al. show through genome analysis that *Verrucomicrobium spinosum* contains a novel open reading frame which is predicted to encode a fusion of the peptidoglycan synthesis enzymes MurB and MurC. The fusion gene was able to complement *Escherichia coli murB* and *murC* mutants and could be identified in specific lineages of the Verrucomicrobium phylum. Rast et al. describe three novel strains belonging to a novel genus of Verrucomicrobia subdivision 4 (*Lacunisphaera* gen. nov.) and detect peptidoglycan in their cell walls.

The cell surface can play an important role in the interaction with the environment and other (host) cells. Ottman et al. investigated the outer membrane proteome of Verrucomicrobia *Akkermansia muciniphila*. This is a beneficial member of the human gut microbiome as decreased levels have been associated with diseases. Because surface-exposed molecules play an important role in colonization and communication with the host and other microorganisms, the authors analyzed the outer membrane proteome. They found that the most abundant outer membrane protein PilQ most likely functions as a type IV pili secretin in *A. muciniphila*. van Teeseling et al. characterized the glycosylation of the S-layer which forms the outermost layer of the anammox Planctomycete cell wall. The S-layer is heavily glycosylated with an O-linked oligosaccharide which is additionally modified by methylation.

Finally, the Planctomycetes have various atypical modes of cell division; from FtsZ-less binary fission to FtsZ-less budding. Rivas-Marín et al. review the lack and presence of peptidoglycan in PVC bacteria and its involvement in chlamydial cell division. They also hypothesize about the possible evolution of the different modes of PVC cell division.

## Chlamydiae

Obligate intracellular Chlamydia are important pathogens of terrestrial and marine vertebrates. However, pathogenesis and host specificity are still largely unknown. Fehr et al. developed the first larval zebrafish model for chlamydial infections with *Waddlia chondrophila*. *Waddliacea* can infect and replicate in epithelia and macrophages. They demonstrate that *W. chondrophila* is taken up and replicates in phagocytic cells (neutrophils) as well as macrophages and that myeloid differentiation factor 88 (My88) mediated signaling plays a role in the innate immune reaction to *W. chondrophila*. Seth-Smith et al. analyse genomes and ultrastructure of as-of-yet uncultivated chlamydial pathogens (Ca. Similichlamydiaceae) that cause epitheliocystis directly from tissue of gilthead seabream (*Sparus aurata*). They show that infection by chlamydial inclusions develops in a perinuclear location and follows a developmental cycle of replicating bodies and elementary bodies.

## Genetic tools

For a long time Planctomycete research was hampered by the lack of genetic tools. However, recently the first genetic tools were developed for the Planctomycete *Planctopirus limnophila* (Jogler et al., 2011; Schreier et al., 2012; Erbilgin et al., 2014). Here, Rivas-Marín et al. developed genetic tools (mutagenesis by homologous recombination) for *P. limnophila* and three other Planctomycetes: *G*. *obscuriglobus, Gimesia maris*, and *Blastopirellula marina*.

## Future directions

The future directions for PVC research will most likely include important topics like; understanding their environmental significance, exploring the impact on and potential in human health and biotechnology, interaction with hosts, and other (micro)organisms, developing more genetic tools in more species, understanding their unusual cell division and in some cases life cycles and finally re-evaluate the PVC phylogeny and classification.

## Author contributions

All authors listed have made a substantial, direct, and intellectual contribution to the work, and approved it for publication.

### Conflict of interest statement

The authors declare that the research was conducted in the absence of any commercial or financial relationships that could be construed as a potential conflict of interest.
